# Associations between physician home visits for the dying and place of death: A population-based retrospective cohort study

**DOI:** 10.1371/journal.pone.0191322

**Published:** 2018-02-15

**Authors:** Peter Tanuseputro, Sarah Beach, Mathieu Chalifoux, Walter P. Wodchis, Amy T. Hsu, Hsien Seow, Douglas G. Manuel

**Affiliations:** 1 Bruyère Research Institute, Ottawa, Ontario, Canada; 2 Ottawa Hospital Research Institute, Clinical Epidemiology Program, Ottawa, Ontario, Canada; 3 Department of Medicine, Division of Palliative Care, University of Ottawa, Ottawa, Ontario, Canada; 4 Institute for Clinical Evaluative Sciences, Population Health and Primary Care, Ottawa, Ontario, Canada; 5 Institute for Health Policy, Management & Evaluation, University of Toronto, Ontario, Canada; 6 Department of Oncology, McMaster University, Hamilton, Ontario, Canada; National Institute of Health, ITALY

## Abstract

**Background:**

While most individuals wish to die at home, the reality is that most will die in hospital.

**Aim:**

To determine whether receiving a physician home visit near the end-of-life is associated with lower odds of death in a hospital.

**Design:**

Observational retrospective cohort study, examining location of death and health care in the last year of life.

**Setting/Participants:**

Population-level study of Ontarians, a Canadian province with over 13 million residents. All decedents from April 1, 2010 to March 31, 2013 (n = 264,754)

**Results:**

More than half of 264,754 decedents died in hospital: 45.7% died in an acute care hospital and 7.7% in complex continuing care. After adjustment for multiple factors–including patient illness, home care services, and days of being at home–receiving at least one physician home visit from a non-palliative care physician was associated with a 47% decreased odds (odds-ratio, 0.53; 95%CI: 0.51–0.55) of dying in a hospital. When a palliative care physician specialist was involved, the overall odds declined by 59% (odds ratio, 0.41; 95%CI: 0.39–0.43). The same model, adjusting for physician home visits, showed that receiving palliative home care was associated with a similar reduction (odds ratio, 0.49; 95%CI: 0.47–0.51).

**Conclusion:**

Location of death is strongly associated with end-of-life health care in the home. Less than one-third of the population, however, received end-of-life home care or a physician visit in their last year of life, revealing large room for improvement.

## Introduction

Death is certain, but the circumstances surrounding death are not. When given the choice, most people prefer to die at home.[1–4] Although there is a gradual shift towards home deaths in countries such as the United Kingdom and United States[5,6], most deaths still happen in hospital[4, 6–11]; one recent review of 45 studies from 36 primarily developed countries confirmed this finding.[12] Location of death not only impacts the dying experience, but also impacts healthcare utilization and costs.[11, 13] End-of-life care consumes a significant portion of overall health care expenditure[14–16], primarily attributable to a rise in inpatient care in the last weeks of life[14,17]; reducing hospital deaths would reduce this cost. While most who are dying are cared for by a physician, it is unclear if receiving a home visit near the end of life–a declining staple of palliative care in many jurisdictions[18]–effectively enables people to die in the community.

Location of death is influenced by complex interactions between illness, individual factors, and environmental factors[19]. It is influenced by the socioeconomic characteristics of individuals, their functional and care needs, and their support networks; it is also affected by the availability of community and facility based healthcare services.[4,20] Receiving community palliative care earlier in the disease trajectory, for example, has been shown to increase the patient’s likelihood of dying at home.[21]

To facilitate care and death in the community, home visits near the end-of-life are conducted by primary care physicians, palliative care physicians, nurses and other health professionals.[22] In our study setting–Ontario, Canada–publicly funded home care is delivered through regional Local Health Integration Networks (LHIN), which mainly provides nursing, personal support worker, and therapist supports to help patients deal with their disability. Ontarians receive universal coverage from the government for health services deemed to be essential. Referral to the LHIN can be made by a health care provider, patients, or their caregivers. Physician home visits are typically conducted separately by primary care physicians who provide such care under their own volition (i.e., outside of a structured hospice system) for their own patients. The exception is that there are specialist palliative care physicians–often working under a regional model–who take-over or share the palliative care of dying patients, identified often through cancer centers, post-discharge from acute care, or by a patient’s family physician. Like most jurisdictions internationally, there is no systematic home hospice system throughout the province, unlike what might be found in the United States or United Kingdom.[23–25]

Support is provided during the visits, and also through subsequent phone/on-call coverage. These services aim to improve the quality of life remaining, including reducing the likelihood of hospital visits near the end-of-life. Apart from the lack of a consistent system, there are, however, many barriers to providing physician home visits, including lack of time and remuneration, questions about effectiveness, deficits in palliative care training, and discomfort with providing care outside of the office setting.

At least seven studies have examined the association between physician home visits and place of death, with all suggesting an increase in the likelihood of home deaths.[26–32] There are, however, limitations to these studies, hampering the overall strength of the evidence and the ability to make policy recommendations. Five of the studies, for example, examined only cancer patients (n = 92 to 4,092 deaths)[28–32], and the two that did not (n = 216 and 295) consisted only of decedents receiving home care.[26,27] None examined all deaths at a population level, and none controlled for a full set of potentially influential confounders, including level of morbidity and the concurrent delivery of home care. Furthermore, previous studies did not address potential indication bias; that those receiving home visits are necessarily at home, and that those at home (especially near the end-of-life) in turn have a higher chance of dying at home.

We address the limitations of previous studies. This large, population-level study (n = 264,754 deaths) examines the separate associations between physician home visits and home care near the end-of-life and location of death, controlling for patient socio-demographics, health, and location of care.

## Methods

We conducted an observational, retrospective cohort study examining location of death of decedents in Ontario, Canada. We captured all deaths in a 3-year period, from April 1, 2010 to March 31, 2013. Individuals dying in an acute care hospital, a complex continuing care hospital, or rehabilitation facilities were classified as having death in a hospital.

### Data sources

Encrypted health card numbers were used as unique identifiers and linked across several administrative databases held at the Institute for Clinical Evaluative Sciences (ICES). All data were de-identified and anonymized. Ethics approval was obtained from the Sunnybrook Health Sciences Centre Research Ethics Board in Toronto, Canada and from the Ottawa Health Science Network Research Ethics Board in Ottawa, Canada.

Deaths were captured from the Registered Persons Database (RPDB). Location of death in acute care, complex continuing care, and rehabilitation facilities were identified through the Canadian Institute for Health Information Discharge Abstract Database, the Continuing Care Reporting System (CCRS), and the National Rehabilitation Reporting System, respectively. Any deaths not captured in one of these databases were considered to be a death outside of a hospital. This included deaths in nursing homes (CCRS) and deaths in hospices.

### Population characteristics

The age and sex of decedents were captured in the RPDB, along with the postal code of residence at death. Following well established methods, both neighborhood income and rurality were captured by linking to Statistics Canada census data using postal codes. The presence of chronic conditions at death were captured using previously developed–and in some cases validated–chronic disease databases at ICES.[33] As a measure of multi-morbidity, the Johns Hopkins Aggregated Diagnosis Groups (ADGs) were used to generate ADG Scores[34], shown to be predictive of mortality.

### Health services near the end-of-life

Home care services were captured using the Resident Assessment Instrument–Home Care databases. Palliative home care was captured when a patient was given an end-of-life designation, which allows them to access additional and often specialized palliative care services. Any subsequent services provided for patients under this designation were recorded as being palliative in nature.

Physician home visits in the last year of life were identified through physician billing claims for services delivered at home, captured in the Ontario Health Insurance Plan database (codes used in S1 File). We captured the subset of home visits delivered by palliative care physician specialists, identified using a previously validated definition of greater than 10% of all billings classified as palliative care.[35]

### Analysis

A logistic regression model was constructed for the primary outcome of death in a hospital. The primary intervention of interest was a physician home visit in the last year of life. We also examined the effect of a palliative care specialist being involved in at least one of the visits. The secondary exposure of interest was the involvement of publicly funded home care; specifically for those receiving an end-of-life designation prior to death.

The multivariable model controlled for age, sex, neighborhood income quintile, rurality, year of death, rostering to a family physician, and ADG Score. We also adjusted for the number of days that a decedent was at home in the last 30 days prior to death since decedents must be at home to receive a home visit (the exposure). A decedent who is at home close to death (i.e., in the last 30 days prior to death) in turn has a higher chance of dying in their home (the outcome); thus not at home close to death is a potential confounder for the relationship between home visits and dying in the home. Finally, we additionally controlled for whether or not a decedent was home 1 week prior to death (binary variable: yes/no), following the observation that the effect of home visit on home deaths steeply rose for visits occurring around this time period. All analyses were conducted using SAS 9.3 (SAS Institute Inc., Cary, NC), and statistical significance was set at p<0.05.

#### Sensitivity analyses

To further explore "being at home" as a potential confounder, we performed two sets of sensitivity analyses. *First*, on each day prior to death, we examined only those who are at home (i.e., not in hospital) on that day. We then separated those who have a physician visit and those who did not, by day prior to death. For each of those two groups, we examined the proportion that died in a hospital. *Second*, we re-ran the multivariable model, separately excluding home visits 30 and 7 days before death, while removing the now redundant variables number of days home in last 30 days and being home at 7 days prior to death.

*Last*, we excluded decedents with a long-term care stay (also known as nursing homes) in their last 30 days of life. Long-term care residents typically receive in-house physician care, but may still receive home visits from other physicians (e.g., their family physician). One could expect that the incremental effect of physician home visits, in an already medicalized environment (e.g., all have 24-hour nursing care), would be dampened.

## Results

**Fig 1:**
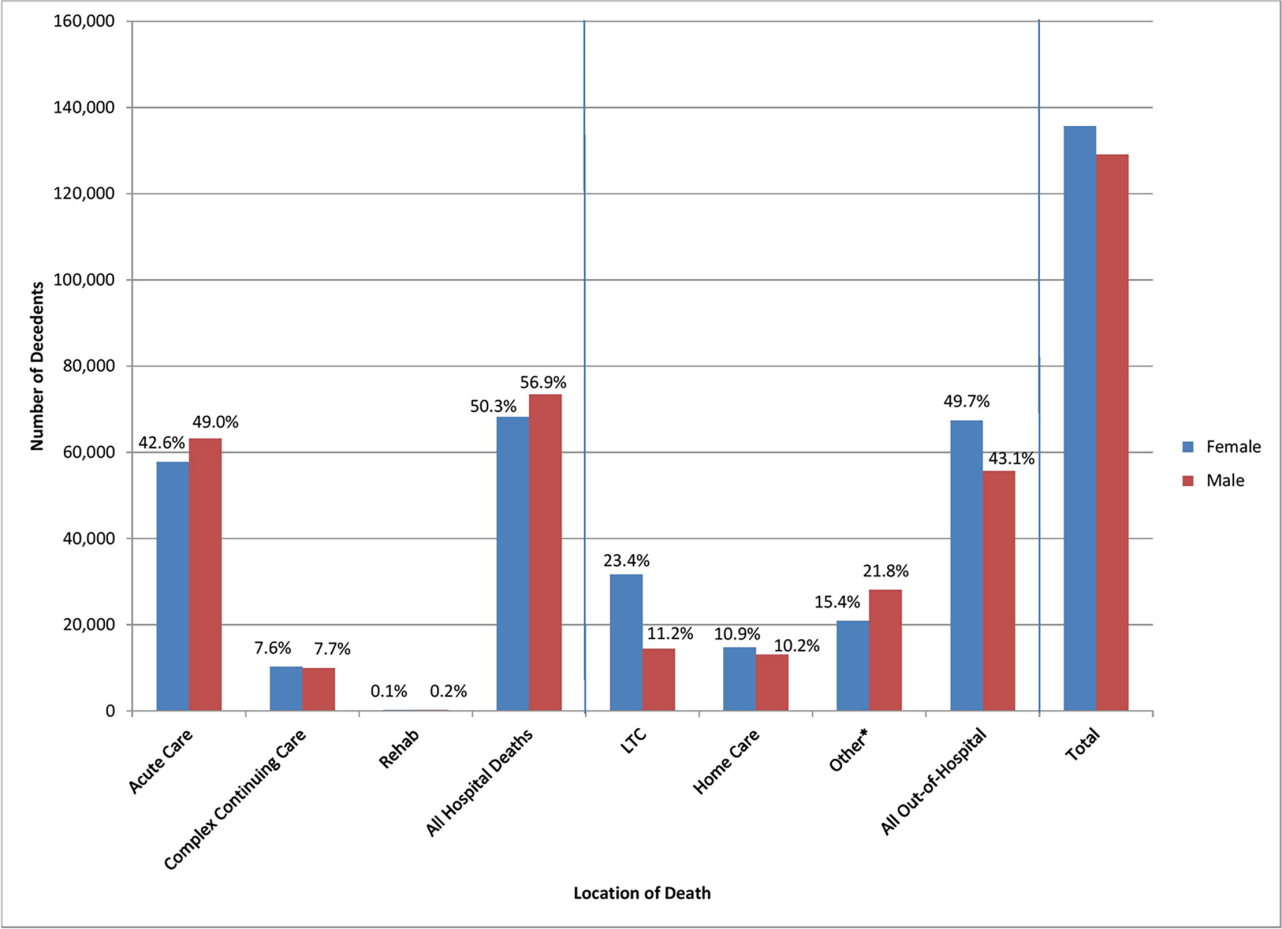
Location of death of 264,754 decedents by sex, 2010/11-2012/13.

We captured 264,754 deaths between fiscal years 2010/11 and 2012/13. Over half of decedents (53.5%) died in a hospital, and the majority in an acute care setting (45.7%) (Fig 1). More than double the proportion of female decedents died in long-term care facilities compared to their male counterparts. Excluding deaths in long-term care, 26.3% of females and 31.9% of males died in the community, including hospices and assisted living facilities.

### Population characteristics

Table 1 captures the characteristics of decedents dying in and outside of hospital. The proportion dying in a hospital peaks between the ages 55–84 years. Those living in higher income quintile and rural neighborhoods tend to die more often in the community. An equally high proportion (61.9%) of those dying with cancer, chronic obstructive pulmonary disease, and congestive heart failure died in a hospital; conversely, only 39.1% of those with dementia died in hospital, as a large proportion died in long-term care facilities. Increasing ADG Scores were strongly associated with a hospital death. Overall rates of hospital deaths have been slowly declining from 54.4% in 2010 to 51.9% in 2013.

**Table 1:** Characteristics of 264,754 decedents, according to location of death.

	Death in Hospital^a^ (%)		Death Out-of-Hospital (%)		Total (column %)	
**Age (at death)**						
<19	1,894	(48.2%)	2,032	(51.8%)	3,926	(1.5%)
19–44	3,114	(39.1%)	4,856	(60.9%)	7,970	(3.0%)
45–54	7,166	(51.5%)	6,747	(48.5%)	13,913	(5.3%)
55–64	15,738	(56.4%)	12,165	(43.6%)	27,903	(10.5%)
65–74	25,936	(60.4%)	17,017	(39.6%)	42,953	(16.2%)
75–84	44,021	(59.0%)	30,570	(41.0%)	74,591	(28.2%)
85–94	38,541	(49.3%)	39,565	(50.7%)	78,106	(29.5%)
95+	5,253	(34.1%)	10,139	(65.9%)	15,392	(5.8%)
**Sex**						
Female	68,235	(50.3%)	67,399	(49.7%)	135,634	(51.2%)
Male	73,428	(56.9%)	55,692	(43.1%)	129,120	(48.8%)
**Income quintile**						
Missing	1,833	(49.3%)	1,883	(50.7%)	3,716	(1.4%)
Lowest	32,833	(54.7%)	27,232	(45.3%)	60,065	(22.7%)
Low	30,418	(55.5%)	24,426	(44.5%)	54,844	(20.7%)
Middle	26,792	(53.1%)	23,679	(46.9%)	50,471	(19.1%)
High	25,787	(52.4%)	23,425	(47.6%)	49,212	(18.6%)
Highest	24,000	(51.7%)	22,446	(48.3%)	46,446	(17.5%)
**Rurality**						
Missing	1,179	(47.0%)	1,332	(53.0%)	2,511	(0.9%)
Urban	120,580	(53.8%)	103,399	(46.2%)	223,979	(84.6%)
Rural	19,904	(52.0%)	18,360	(48.0%)	38,264	(14.5%)
**Year of Death**						
2010	35,145	(54.4%)	29,453	(45.6%)	64,598	(24.4%)
2011	47,083	(53.9%)	40,334	(46.1%)	87,417	(33.0%)
2012	46,707	(52.9%)	41,529	(47.1%)	88,236	(33.3%)
2013	12,728	(51.9%)	11,775	(48.1%)	24,503	(9.3%)
**Chronic conditions (at death)**						
Cancer	71,160	(61.9%)	43,739	(38.1%)	114,899	(43.4%)
COPD	40,902	(61.9%)	25,181	(38.1%)	66,083	(25.0%)
CHF	55,014	(61.9%)	33,847	(38.1%)	88,861	(33.6%)
Dementia	29,476	(39.1%)	45,880	(60.9%)	75,356	(28.5%)
Depression	28,896	(53.4%)	25,204	(46.6%)	54,100	(20.4%)
Diabetes	54,703	(58.7%)	38,432	(41.3%)	93,135	(35.2%)
Renal Disease	43,175	(68.6%)	19,758	(31.4%)	62,933	(23.8%)
Stroke	24,683	(59.7%)	16,658	(40.3%)	41,341	(15.6%)
**ADG Score Quintiles**^b^						
Lowest	11,554	(21.6%)	41,926	(78.4%)	53,480	(20.2%)
Low	26,852	(51.4%)	25,419	(48.6%)	52,271	(19.7%)
Middle	29,607	(59.5%)	20,126	(40.5%)	49,733	(18.8%)
High	36,331	(64.8%)	19,710	(35.2%)	56,041	(21.2%)
Highest	37,319	(70.1%)	15,910	(29.9%)	53,229	(20.1%)

^a^Hospitals: Acute care, complex continuing care, and rehabilitation hospitals

^b^Adjusted Diagnosis Groups (ADG) Scores are calculated using past previous health care use, indicative of multi-morbidity and associated with an increase in mortality.[34]

### Health services near the end-of-life

#### Home care

About 3 out of 5 decedents (60.7%) received home care in the last year of life, 30.2% of whom (18.4% overall) received an end-of-life designation prior to death (Table 2). High proportions of those receiving home care died in hospital (Table 2). Among those receiving home care, however, those that received an end-of-life designation had a much higher chance of dying in the community.

**Table 2:** Location of death, according to whether home care or physician home visits were received prior to death.

Service Received	Death in Hospital^c^ (%)		Death Out-of-Hospital (%)		Total (Column %)	
**Home care within last 30 days of life**						
Yes–Any designation	66,213	(59.5)	44,994	(40.5)	111,207	(42.0)
Yes–End-of-life designation^a^	17,440	(40.8)	25,290	(59.2)	42,730	(16.1)
No	75,450	(49.1)	78,097	(50.9)	153,547	(58.0)
**Home care within last 90 days of life**						
Yes–Any designation	81,690	(60.9)	52,538	(39.1)	134,228	(50.7)
Yes–End-of-life designation^a^	20,070	(43.1)	26,544	(56.9)	46,614	(17.6)
No	59,973	(46.0)	70,553	(54.1)	130,526	(49.3)
**Home care within last year of life**						
Yes–Any designation	94,865	(59.0)	65,928	(41.0)	160,793	(60.7)
Yes–End-of-life designation^a^	21,304	(43.9)	27,279	(56.2)	48,583	(18.4)
No	46,798	(45.0)	57,163	(55.0)	103,961	(39.3)
**Physician home visits within last year of life**						
None	120,574	(57.2)	90,147	(42.8)	210,721	(79.6)
Yes—Without palliative physician	13,708	(41.8)	19,088	(58.2)	32,796	(12.4)
Yes— With palliative physician^b^	7,381	(34.8)	13,856	(65.2)	21,237	(8.0)
**Number of physician home visits in last year of life**						
None	120,574	(57.2)	90,147	(42.8)	210,721	(79.6)
1	8,860	(42.4)	12,060	(57.7)	20,920	(7.9)
2	3,879	(42.1)	5,337	(57.9)	9,216	(3.5)
3–4	3,637	(38.4)	5,839	(61.6)	9,476	(3.6)
5–6	1,722	(36.2)	3,038	(63.8)	4,790	(1.8)
7+	2,991	(31.0)	6,670	(69.0)	9,661	(3.7)
**Last date of physician home visit**						
None	120,574	(57.2)	90,147	(42.8)	210,721	(79.6)
<1 week before death	2,991	(11.3)	23,552	(88.7)	26,543	(10.0)
1–2 weeks before death	2,689	(58.6)	1,903	(41.4)	4,592	(1.7)
2–4 weeks before death	3,818	(68.3)	1,770	(31.7)	5,588	(2.1)
4–12 weeks before death	5,701	(72.1)	2,204	(27.9)	7,905	(3.0)
12+ weeks before death	5,890	(62.6)	3,515	(37.4)	9,405	(3.6)

^a^Signifies those who received an end-of-life designation. Such individuals are often eligible for additional services, including access to a specialist palliative care team in some regions

^b^At least one of the encounters with the patient was made by a palliative physician

^c^Hospitals include acute care hospitals, complex continuing care hospitals, and rehabilitation facilities

#### Physician home visits

About 1 in 5 decedents (20.4%) received a physician home visit in the last year of life, among whom 39.3% received at least one visit from a palliative care physician. The proportion dying in a hospital declined from 57.2% for those without a physician home visit, to 41.8% for those receiving at least one home visit but none from a palliative care specialist, and to 34.8% if a palliative care physician conducted at least one visit. The number of physician home visits was directly associated with declining hospital deaths, from 57.2% for those with no visits, to 31.0% for those with more than 7 visits in the last year of life. A last visit date in the last week prior to death (49.1% of decedents receiving a home visit) was strongly associated with an out-of-hospital death. Those receiving a last visit date outside of the last week of life had a high, albeit unadjusted, rate of hospital deaths.

### Multivariable models for odds of a hospital death

Adjusting for multiple covariates, the odds ratio for un-rostered patients was 1.31 (95% Confidence Interval (CI): 1.28 to 1.34) (Table 3). Those receiving home care had only a slightly higher rate of hospital death, despite the expectation of having greater need for supportive services and being less likely to die unexpectedly outside of hospitals. Those receiving an end-of-life home care designation, however, had an odds ratio of 0.49 (95% CI: 0.47 to 0.51). Similarly, those receiving a physician home visit without and with a palliative care specialist involvement had an odds ratio of 0.53 (95% CI: 0.51 to 0.55) and 0.41 (95% CI: 0.39 to 0.43), respectively.

**Table 3:** Logistic regression for outcome of hospital deaths among 264,754 decedents, 2010/11-2012/13.

	No. of Decedents	Odds Ratio (95% CI) for Hospital Death		P value
**Age**				
<19	3,926	1.00		
19–44	7,970	0.35	(0.31 to 0.39)	**< .001**
45–54	13,913	0.41	(0.36 to 0.45)	**< .001**
55–64	27,903	0.43	(0.39 to 0.48)	**< .001**
65–74	42,953	0.43	(0.39 to 0.48)	**< .001**
75–84	74,591	0.35	(0.32 to 0.39)	**< .001**
85–94	78,106	0.26	(0.23 to 0.28)	**< .001**
95+	15,392	0.19	(0.17 to 0.21)	**< .001**
**Sex**				
Male	135,634		1.00	
Female	129,120	0.92	(0.90 to 0.94)	**< .001**
**Income quintile**				
Lowest	60,065		1.00	
Low	54,844	1.03	(1.00 to 1.06)	0.073
Middle	50,471	0.99	(0.96 to 1.02)	0.485
High	49,212	0.95	(0.92 to 0.98)	**0.002**
Highest	46,446	0.99	(0.95 to 1.02)	0.392
**Rurality of residence**				
Rural resident	38,264		1.00	
Urban resident	223,979	1.00	(0.98 to 1.03)	0.814
**Year of death**				
2010	64,598		1.00	
2011	87,417	0.93	(0.90 to 0.96)	**< .001**
2012	88,236	0.91	(0.88 to 0.93)	**< .001**
2013	24,503	0.90	(0.86 to 0.94)	**< .001**
**Primary care model**				
Rostered	69,752		1.00	
Un-rostered	195,002	1.31	(1.28 to 1.34)	**< .001**
**Home care in last year of life**				
None	103,961		1.00	
Yes–no end-of-life designation	112,210	1.09	(1.06 to 1.12)	**< .001**
Yes–end-of-life designation	48,583	0.49	(0.47 to 0.51)	**< .001**
**Physician home visits in last year of life**				
None	210,721		1.00	
Yes–from non-palliative physician	32,796	0.53	(0.51 to 0.55)	**< .001**
Yes–from palliative physician^a^	21,237	0.41	(0.39 to 0.43)	**< .001**
**Adjusted Diagnosis Group (ADG) Score**				
Continuous Score (-29 to 76)	264,754	1.03	(1.03 to 1.03)	**< .001**
**At home 1 week before death**				
No	96,997		1.00	
Yes	167,757	0.50	(0.48 to 0.52)	**< .001**
**No. days at home in last month of life**				
Continuous (0–30)	264,754	0.84	(0.84 to 0.84)	**< .001**

^a^At least one of the home visits in the last year of life was made by a palliative physician

#### Sensitivity analyses

**Fig 2:**
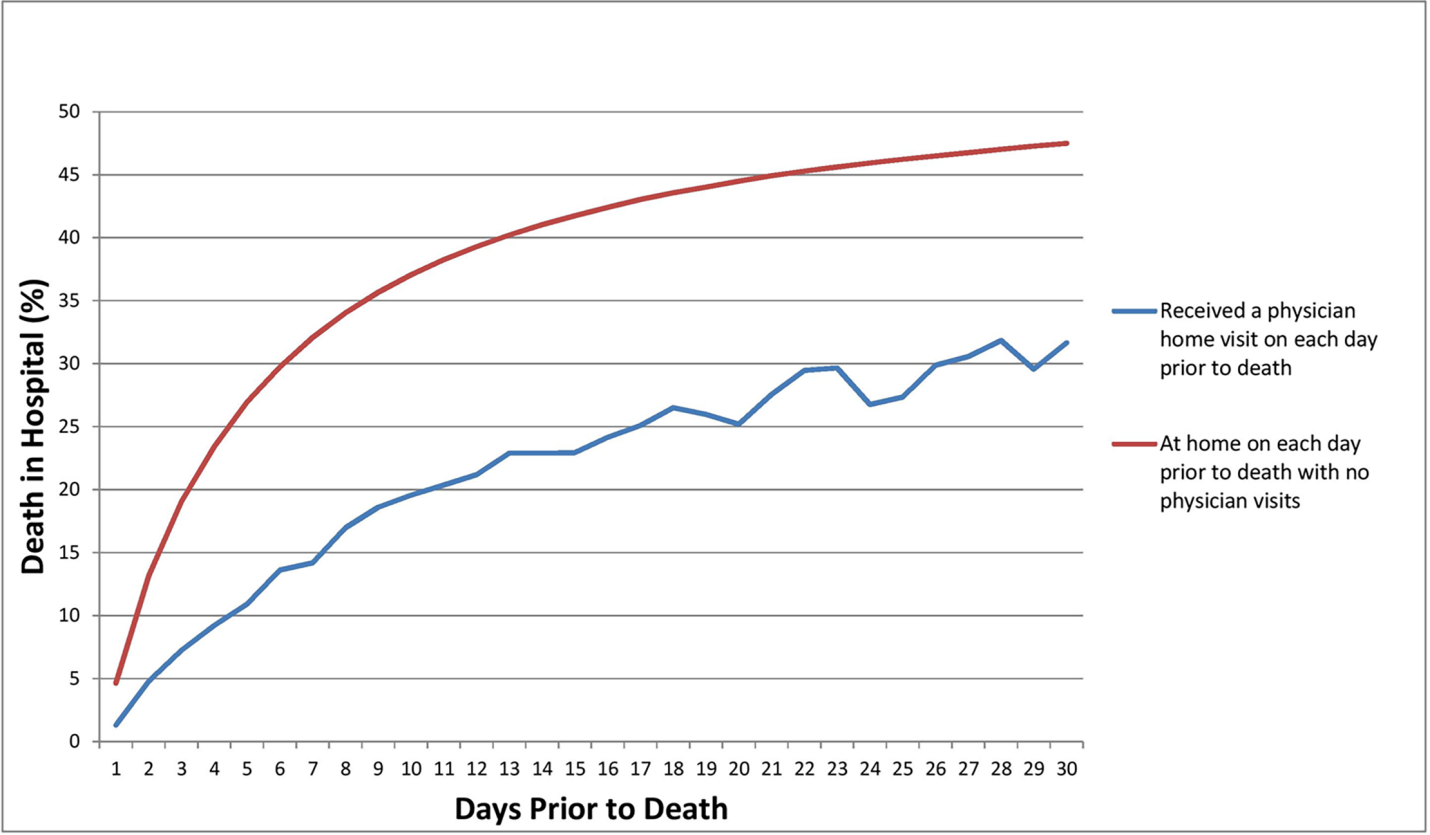
Effect of physician home visits by day of visit prior to death, for decedents who are home (i.e., out-of-hospital) at any given day in the last month prior to death.

The proportion of hospital deaths among those at home and receiving a physician home visit was lower than for those at home and not receiving a home visit for each of the 30 days prior to death (Fig 2). Our second sensitivity analyses found that when physician home visits in the last week of life were excluded, physician visits with and without a palliative care involvement had a slightly attenuated odds ratio of 0.57 (95% CI: 0.55 to 0.59) and 0.79 (95% CI: 0.77 to 0.81), respectively (data not shown). The odds ratios were 0.72 (95% CI: 0.69 to 0.75) and 0.87 (95% CI: 0.84 to 0.90) when physician home visits in the last month of life were excluded.

Finally, excluding long-term care residents augmented the protective effect of home visits on hospital deaths; the odds ratios without and with palliative care physician involvement were 0.37 (95% CI: 0.36 to 0.38) and 0.30 (95% CI: 0.29 to 0.32), respectively (S1 Table).

## Discussion

A good death contributes to a good life lived. We have shown that receiving a physician home visit near the end-of-life is strongly associated with an out-of-hospital death, aligned with the wishes of most.[1–4] Some may argue that the community may not be the best dying environment, given that lack of community supports leads to a large proportion of the population actually dying in hospital. We show indeed that if you increase the level of community supports, you may avoid some (but not all) hospital deaths. We do not make the assertion that all deaths should occur in the community, but rather than many more can be facilitated out-of-hospital. This is true regardless of palliative care physician involvement–which slightly augments the impact on home deaths. We also observed that being recognized formally as being end-of-life by home care services (and likely receiving specialist palliative care in the home) has a similar, *independent* association. We found, however, that only a minority of the population ever received a physician home visit or an end-of-life home care designation–not unlike other jurisdictions[18, 36, 37]–signaling large room for improvement.

Our study addresses limitations of previous studies by conducting analyses adjusted for multiple potential confounders, including disease comorbidity and time spent at home prior to death–both shown in our study to be strongly associated with location of death. Our adjusted odds ratio was less impressive than those observed in previous studies. The largest previous study among cancer patients[29], for example, found that home visits in the last 3 months had an odds ratio of 0.08 for hospital deaths. The differences observed could be due to several reasons, including adjustment for confounders, and differences in the base population and health care system (i.e., intervention) studied; only 1 previous study was conducted in Canada[27], with the others being conducted in Denmark[28, 29, 32] and Japan.[26,30,31].

A strength of our study is the inclusion of a large population-level cohort (more than 30 times larger than all previous studies combined) that allowed us to make robust conclusions about the potential effect of *all* home visits. The inclusion of all deaths, however, can be perceived to have several limitations. First, we *chose* not to exclude deaths from external causes (6.5% of Canadian deaths in 2011)[38], of which many are sudden. It is difficult (and not feasible with administrative data) to identify which deaths can be classified as sudden–enough so that a physician home visit could not be reasonably conducted.[39] Second, palliative care unit (PCU) beds within a hospital setting are poorly tracked in Ontario, and we were thus unable to separate deaths in these settings, likely a small minority of all deaths.[23] It is unclear to what degree physician home visits facilitate deaths in a PCU bed (i.e., when symptoms are unmanageable at home), or prevent hospitalizations that lead to death in a PCU. Lastly, we have shown that we are attenuating the protective effect of home visits by including deaths for long-term care residents, who already lived in a medicalized setting with 24 hour nursing care and often an on-call physician.

A challenge to any study examining the relationship between home visits and location of death is controlling for the confounder of "being at home", a necessity of receiving a home visit, and related to home deaths. We attempted to address this in our multivariable models. Additionally, we conducted sensitivity analyses to show the consistency of our results. We first studied only people at home (i.e., out-of-hospital) on each day in the last 30 days of life, and showed that home visits reduce the probability of a hospital death, regardless of the day of the visit (Fig 2). We also showed that removing home visits close to death (1 week and 30 days prior to death) still resulted in significant, albeit attenuated, reductions in the odds of a hospital death.

The relationship between physician home visits and place of death is complex, influenced by the characteristics of the patient, the level of supports at home, and health system factors such as the mix of available hospital and community services.[26–32] We adjusted for many, but not all, of these variables. Notably, we could not consider the presence of a caregiver in the home, since we did not have access to such data at a population level. Nevertheless, this study provides insight and impetus for future intervention studies–with ideally a randomized trial design–that would further reduce potential biases. In the absence of such studies, however, the low rates of physician home visits (20.4%) and palliative home care (18.4%) among our decedent population, coupled with the high rates of hospital deaths and the body of evidence supporting the association between home supports and home deaths all point to the need for initiatives to improve the reach of such care.

### Conclusions

We have shown with *consistency* (i.e., by physician type, by timing of visit, by patient population, etc.) that home visits are strongly associated with a community death. A person with both a physician home visit and palliative home care designation had an even larger reduction in odds of a hospital death (73% and 76% without/with palliative care physician–data not shown). Physician home visits exhibited a *dose-response* association between number of visits and odds of a hospital death. These observations are *plausible* and intuitive–regardless of jurisdiction; a simple illustration is a person with pain or agitation who will often, at one point prior to death, be unable to ingest medications orally. Without physician care in the community, this person would unlikely have access to subcutaneous or intravenous palliative care medications, and will likely visit the emergency room in a crisis, where treatment (and not palliation) of underlying conditions is often the default option. This subsequently leads to an admission and death in hospital, as transfers back into the community at the end-of-life are difficult.

An involved physician in the home may also facilitate access to additional services–including home care–involving allied health care practitioners and, at times, a specialist palliative care team. The availability of such teams (often including a specialist palliative care physician), however, is too limited to provide care to the majority of the population in most jurisdictions, including Canada.[40, 41] In our study, palliative care specialists reach less than 10% of decedents in their home. This gap can be filled by non-palliative care specialists, who we have shown to have similar effectiveness in reducing hospital deaths. Filling this gap requires addressing a complex web of barriers including training, remuneration, and early identification of palliative care patients. With most decedents never receiving a home visit by any physician prior to death[18, 36, 37], closing this gap will have large population effects on facilitating desired home deaths, and improving the quality of remaining life.

## Supporting information

S1 STROBE Checklist Checklist of items that should be included in reports of cohort studies. (DOCX)

S1 File Codes used to identify the home based physician visits. (DOCX)

S1 Table Logistic regression for hospital deaths excluding decedents who had a long-term care (i.e., nursing home) stay in their last 30 days of life, Ontario decedents (n = 205,431), fiscal year 2010/11 to 2012/13. (DOCX)
