# Supplementary material for: Associations between physician home visits for the dying and place of death: A population-based retrospective cohort study
Source: PLoS One. 2018 Feb 15;13(2):e0191322. doi: 10.1371/journal.pone.0191322 (PMC5813907; doi:10.1371/journal.pone.0191322)
Supplement: S1 STROBE Checklist — (DOCX) [file pone.0191322.s001.docx]

**S1 STROBE Checklist.** Checklist of items that should be included in reports of ***cohort studies***

|  | Item No | Recommendation |  |  |
| --- | --- | --- | --- | --- |
| **Title and abstract** | 1 | (*a*) Indicate the study’s design with a commonly used term in the title or the abstract | Included in abstract pg. 2 |  |
|  |  | (*b*) Provide in the abstract an informative and balanced summary of what was done and what was found | Pg. 2 |  |
| Introduction | | |  |  |
| Background/rationale | 2 | Explain the scientific background and rationale for the investigation being reported | Included in “Introduction” section Pg. 4-6 |  |
| Objectives | 3 | State specific objectives, including any prespecified hypotheses | Last paragraph of introduction |  |
| Methods | | |  |  |
| Study design | 4 | Present key elements of study design early in the paper | “Methods” Section Pg. 6 |  |
| Setting | 5 | Describe the setting, locations, and relevant dates, including periods of recruitment, exposure, follow-up, and data collection | “Methods” Section Pg. 6 |  |
| Participants | 6 | (*a*) Give the eligibility criteria, and the sources and methods of selection of participants. Describe methods of follow-up | “Methods” Section Pg. 6 |  |
|  |  | (*b*) For matched studies, give matching criteria and number of exposed and unexposed | n/a |  |
| Variables | 7 | Clearly define all outcomes, exposures, predictors, potential confounders, and effect modifiers. Give diagnostic criteria, if applicable | Throughout methods and in S1 File |  |
| Data sources/ measurement | 8* | For each variable of interest, give sources of data and details of methods of assessment (measurement). Describe comparability of assessment methods if there is more than one group | “Data sources” section Pg. 6-7 |  |
| Bias | 9 | Describe any efforts to address potential sources of bias | “Analysis” section Pg. 8 |  |
| Study size | 10 | Explain how the study size was arrived at | “Methods” section Pg. 6 |  |
| Quantitative variables | 11 | Explain how quantitative variables were handled in the analyses. If applicable, describe which groupings were chosen and why | “Analysis” section Pg. 8 |  |
| Statistical methods | 12 | (*a*) Describe all statistical methods, including those used to control for confounding | “Analysis” section Pg. 9 |  |
|  |  | (*b*) Describe any methods used to examine subgroups and interactions | “Sensitivity analyses” section Pg. 9 |  |
|  |  | (*c*) Explain how missing data were addressed | Missing data included in tables |  |
|  |  | (*d*) If applicable, explain how loss to follow-up was addressed | n/a |  |
|  |  | (*e*) Describe any sensitivity analyses | “Sensitivity analyses” section Pg. 9 |  |
| Results | | |  |  |
| Participants | 13* | (a) Report numbers of individuals at each stage of study—eg numbers potentially eligible, examined for eligibility, confirmed eligible, included in the study, completing follow-up, and analysed | “Results” section Pg. 9 |  |
|  |  | (b) Give reasons for non-participation at each stage | n/a |  |
|  |  | (c) Consider use of a flow diagram | Not included |  |
| Descriptive data | 14* | (a) Give characteristics of study participants (eg demographic, clinical, social) and information on exposures and potential confounders | “Population Characteristics” section Pg. 10; Table 1 |  |
|  |  | (b) Indicate number of participants with missing data for each variable of interest | Table 1 |  |
|  |  | (c) Summarise follow-up time (eg., average and total amount) | n/a |  |
| Outcome data | 15* | Report numbers of outcome events or summary measures over time | Table 2 |  |
| Main results | 16 | (*a*) Give unadjusted estimates and, if applicable, confounder-adjusted estimates and their precision (eg, 95% confidence interval). Make clear which confounders were adjusted for and why they were included | “Results” section Pg. 9-15; Table 3; S1 File |  |
|  |  | (*b*) Report category boundaries when continuous variables were categorized | Displayed throughout tables |  |
|  |  | (*c*) If relevant, consider translating estimates of relative risk into absolute risk for a meaningful time period | n/a |  |
| Other analyses | 17 | Report other analyses done—eg analyses of subgroups and interactions, and sensitivity analyses | “Results” section Pg. 9-15 |  |
| Discussion | | |  |  |
| Key results | 18 | Summarise key results with reference to study objectives | Pg. 15-16 |  |
| Limitations | 19 | Discuss limitations of the study, taking into account sources of potential bias or imprecision. Discuss both direction and magnitude of any potential bias | “Discussion” section Pg. 16 |  |
| Interpretation | 20 | Give a cautious overall interpretation of results considering objectives, limitations, multiplicity of analyses, results from similar studies, and other relevant evidence | “Conclusions” section Pg. 18-19 |  |
| Generalisability | 21 | Discuss the generalisability (external validity) of the study results | “Conclusions” section Pg. 18-19 |  |
| Other information | | |  |  |
| Funding | 22 | Give the source of funding and the role of the funders for the present study and, if applicable, for the original study on which the present article is based | Included in Statements Pg. 22 |  |

*Give information separately for exposed and unexposed groups.

**Note:** An Explanation and Elaboration article discusses each checklist item and gives methodological background and published examples of transparent reporting. The STROBE checklist is best used in conjunction with this article (freely available on the Web sites of PLoS Medicine at http://www.plosmedicine.org/, Annals of Internal Medicine at http://www.annals.org/, and Epidemiology at http://www.epidem.com/). Information on the STROBE Initiative is available at http://www.strobe-statement.org.
