# Supplementary material for: Associations between physician home visits for the dying and place of death: A population-based retrospective cohort study
Source: PLoS One. 2018 Feb 15;13(2):e0191322. doi: 10.1371/journal.pone.0191322 (PMC5813907; doi:10.1371/journal.pone.0191322)
Supplement: S1 File — (DOCX) [file pone.0191322.s002.docx]

**S1 File.** **Codes used to identify the home based physician visits**

Home based physician visits were captured in the Ontario Health Insurance Plan (OHIP) billing database through the following codes:

- An assessment by a physician, rendered in a patient’s home that satisfies, at a minimum, all of the requirements of an intermediate assessment - A901
- Travel billing codes for visits to patient’s home for any reason - B960, B961, B962, B963, B964
- Travel billing code for palliative care home visit - B966
- First person seen billing premium for visits to patient’s home for any reason - B990, B992, B993, B994, B996
- First person seen billing premium for palliative care home visit - B998
