# Supplementary material for: Associations between physician home visits for the dying and place of death: A population-based retrospective cohort study
Source: PLoS One. 2018 Feb 15;13(2):e0191322. doi: 10.1371/journal.pone.0191322 (PMC5813907; doi:10.1371/journal.pone.0191322)
Supplement: S1 Table — (DOCX) [file pone.0191322.s003.docx]

**S1 Table.** Logistic regression for hospital deaths excluding decedents who had a long-term care (i.e., nursing home) stay in their last 30 days of life, Ontario decedents (n = 205,431), fiscal year 2010/11 to 2012/13

|  | **No. of Decedents** | **Odds Ratio (95% CI) for Hospital Death** | | **P value** |
| --- | --- | --- | --- | --- |
| **Age** |  |  |  |  |
| <19 | 3,926 | 1.00 | |  |
| 19-44 | 7,909 | 0.35 | (0.31 to 0.39) | **<.001** |
| 45-54 | 13,672 | 0.41 | (0.37 to 0.46) | **<.001** |
| 55-64 | 26,861 | 0.46 | (0.41 to 0.51) | **<.001** |
| 65-74 | 39,400 | 0.50 | (0.45 to 0.56) | **<.001** |
| 75-84 | 58,972 | 0.52 | (0.47 to 0.58) | **<.001** |
| 85-94 | 47,813 | 0.51 | (0.46 to 0.57) | **<.001** |
| 95+ | 6,878 | 0.48 | (0.42 to 0.54) | **<.001** |
| **Sex** |  |  |  |  |
| Female | 96,175 |  | 1.00 |  |
| Male | 109,256 | 1.09 | (1.07 to 1.12) | **<.001** |
| **Income quintile** |  |  |  |  |
| Lowest | 45,878 |  | 1.00 |  |
| Low | 43,414 | 0.99 | (0.95 to 1.03) | 0.531 |
| Middle | 38,924 | 0.99 | (0.95 to 1.03) | 0.543 |
| High | 37,916 | 0.95 | (0.92 to 0.99) | **0.016** |
| Highest | 35,959 | 0.99 | (0.95 to 1.03) | 0.642 |
| **Rurality of residence** |  |  |  |  |
| Rural resident | 173,819 |  | 1.00 |  |
| Urban resident | 29,182 | 1.07 | (1.04 to 1.11) | 0.967 |
| **Year of death** |  |  |  |  |
| 2010 | 50,256 |  | 1.00 |  |
| 2011 | 67,667 | 0.92 | (0.89 to 0.95) | **<.001** |
| 2012 | 68,739 | 0.89 | (0.86 to 0.92) | **<.001** |
| 2013 | 18,769 | 0.88 | (0.83 to 0.92) | **<.001** |
| **Primary care model** |  |  |  |  |
| Rostered | 48,224 |  | 1.00 |  |
| Un-rostered | 157,207 | 1.12 | (1.09 to 1.15) | **<.001** |
| **Home care in last year of life** |  |  |  |  |
| None | 72,185 |  | 1.00 |  |
| Yes – no end-of-life designation | 85,501 | 1.03 | (0.99 to 1.06) | **0.118** |
| Yes– end-of-life designation | 47,745 | 0.36 | (0.34 to 0.37) | **<.001** |
| **Physician home visits in last year of life** |  |  |  |  |
| None | 157,040 |  | 1.00 |  |
| Yes – from non-palliative physician | 28,047 | 0.37 | (0.36 to 0.38) | **<.001** |
| Yes – from palliative physician^a^ | 20,344 | 0.30 | (0.29 to 0.32) | **<.001** |
| **Adjusted Diagnosis Group (ADG) Score** |  |  |  |  |
| Continuous Score (-29 to 76) | 205,431 | 1.04 | (1.04 to 1.04) | **<.001** |
| **At home 1 week before death** |  |  |  |  |
| No | 88,558 |  | 1.00 |  |
| Yes | 116,873 | 0.47 | (0.45 to 0.49) | **<.001** |
| **No. days at home in last month of life** |  |  |  |  |
| Continuous (0-30) | 205,431 | 0.85 | (0.84 to 0.85) | **<.001** |

## ^a^At least one of the home visits in the last year of life was made by a palliative physician
